# Cohesive network reconfiguration accompanies extended training

**DOI:** 10.1002/hbm.23699

**Published:** 2017-06-24

**Authors:** Qawi K. Telesford, Arian Ashourvan, Nicholas F. Wymbs, Scott T. Grafton, Jean M. Vettel, Danielle S. Bassett

**Affiliations:** ^1^ Department of Bioengineering University of Pennsylvania Philadelphia Pennsylvania 19104; ^2^ Human Research and Engineering Directorate U.S. Army Research Laboratory Aberdeen Maryland 21001; ^3^ Department of Neurology Johns Hopkins University Baltimore Maryland 21218; ^4^ Department of Psychological and Brain Sciences University of California Santa Barbara California 93106; ^5^ Department of Electrical and Systems Engineering University of Pennsylvania Philadelphia Pennsylvania 19104

**Keywords:** connectomics, motor learning, dynamic networks, graph theory, functional magnetic resonance imaging

## Abstract

Human behavior is supported by flexible neurophysiological processes that enable the fine‐scale manipulation of information across distributed neural circuits. Yet, approaches for understanding the dynamics of these circuit interactions have been limited. One promising avenue for quantifying and describing these dynamics lies in multilayer network models. Here, networks are composed of nodes (which represent brain regions) and time‐dependent edges (which represent statistical similarities in activity time series). We use this approach to examine functional connectivity measured by non‐invasive neuroimaging techniques. These multilayer network models facilitate the examination of changes in the pattern of statistical interactions between large‐scale brain regions that might facilitate behavior. In this study, we define and exercise two novel measures of network reconfiguration, and demonstrate their utility in neuroimaging data acquired as healthy adult human subjects learn a new motor skill. In particular, we identify putative functional modules in multilayer networks and characterize the degree to which nodes switch between modules. Next, we define cohesive switches, in which a set of nodes moves between modules together as a group, and we define disjoint switches, in which a single node moves between modules independently from other nodes. Together, these two concepts offer complementary yet distinct insights into the changes in functional connectivity that accompany motor learning. More generally, our work offers statistical tools that other researchers can use to better understand the reconfiguration patterns of functional connectivity over time. *Hum Brain Mapp 38:4744–4759, 2017*. © **2017 The Authors Human Brain Mapping Published by Wiley Periodicals, Inc.**

## INTRODUCTION

Dynamic network analysis has emerged as an important topic in recent years within the field of neuroimaging. The primary motivation for this type of analysis is driven by the concept that functional relationships in the brain are not static, but in fact change over time [de Zwart et al., 2005; Gonzalez‐Castillo et al., 2014]. Studies assessing dynamic brain networks have focused on understanding how transient properties in signals can affect brain network organization across multiple time scales [Bassett et al., 2013a; Hutchison et al., 2013a; Sakoğlu et al., 2010]. Building on several studies assessing the validity and precision of functional connectivity correlations [Hindriks et al., 2016; Hutchison et al., 2013a; Leonardi and Van De Ville, 2015], recent efforts have extended their focus to examine dynamic brain states and their relationship to cognitive function [Bassett et al., 2013a; Braun et al., 2015; Hutchison et al., 2013b]. These studies have demonstrated that dynamic network analysis can uncover time‐dependent changes related to the neurophysiological processes underlying cognition.

One particularly appealing approach for examining dynamic brain states is to study changes in community structure. Community structure describes the pattern of interconnected clusters in a network; groups of nodes that share more connections with other nodes in their group than they do to nodes in other groups are considered communities (or modules) [Fortunato, 2010; Girvan and Newman, 2002]. While community detection algorithms were initially designed for a static network representation, the tools have recently been extended to dynamic networks using a multilayer network formalism [Mucha et al., 2010]. Applying these multilayer network tools to brain networks has demonstrated the presence of communities [Bassett et al., 2011, 2013a; Braun et al., 2015] that map on to well‐known cognitive systems [Bassett et al., 2015] whose interactions with one another change both within a single task [Braun et al., 2015] and across tasks [Cole et al., 2014; Mattar et al., 2015].

A convenient way to study these time‐dependent interactions between communities or modules uses a metric called *network flexibility*, which expresses how often nodes switch communities over time [Bassett et al., 2013a]. Using this measure, one can uncover a temporal core‐periphery structure in which some regions (nodes) in the brain are stably affiliated with their own community (temporal core) and other regions are not stably affiliated with their own community but instead frequently change their affiliation to communities (temporal periphery). The observation of both stable and flexible roles of brain regions in dynamic networks remains robust across different tasks, including motor learning [Bassett et al., 2011], memory recognition [Telesford et al., 2016], and linguistic processing [Chai et al., 2016]. These studies demonstrate that measurements of dynamic networks can uncover transitions between neurophysiological states that underlie cognition.

The measure of network flexibility has offered some important insights into higher‐order cognition in humans. For example, individual differences in network flexibility correlate with individual differences in performance on a *n*‐back working memory task, as well as on the Trails B score [Braun et al., 2015], suggesting its relevance for executive function generally and the psychological construct of cognitive flexibility specifically. Neurophysiological drivers of network flexibility appear to include NMDA, as pharmaco‐fMRI studies demonstrate that an NMDA‐receptor antagonist (Dextromethorphan) can be used to enhance network flexibility in healthy adult human subjects [Braun et al., 2016]. Evidence further suggests that the statistic may be under partial genetic control, as is represents an intermediate phenotype for schizophrenia, a devastating mental disorder that is associated with marked deficits in executive function. Network flexibility demonstrates low values in healthy controls, intermediate values in siblings of people with schizophrenia, and high values in people with schizophrenia [Braun et al., 2016]. Finally, individual differences in network flexibility have been shown to correlate with individual differences in performance on tasks requiring executive function including motor learning [Bassett et al., 2011] and reinforcement learning [Gerraty et al., 2016].

Yet, despite the success of dynamic network methods generally as well as the neurophysiological relevance of network flexibility for executive function specifically, much work still remains to devise metrics of reconfiguration that best capture neurophysiological processes. In particular, in the context of dynamic community detection, the node flexibility probes network dynamics in terms of the frequency of community changes. However, this measure only describes how often changes occur in a system, without giving insight into the nature of those changes. Specifically, the measure is blind to where nodes move when changing community assignment. One might naturally wish to determine whether brain regions change communities in a coordinated manner or whether they change relatively independently from one another. Such changes may illustrate differences in cognitive strategies or neurophysiological drivers, and may also provide insight into heterogeneous differences seen in modular organizations across groups [Stanley et al., 2014].

In this study, we address this methodological challenge by expanding on the formalism of network flexibility. Specifically, we introduce two measures—node cohesion and node disjointedness—that describe distinct types of changes in community structure relevant for higher‐order cognitive function broadly, and learning specifically. The first, node cohesion, measures the degree to which nodes move together (mutually) from one community to another. This metric is particularly appropriate for determining collective changes in the coordinated function of brain regions thought to be a marker of changes in cognitive process or strategy as humans move from early to late learning [Fatima et al., 2016]. The second, node disjointedness, measures the degree to which nodes move separately (independently) from their community to other communities. This metric is particularly appropriate for assessing local processes that appear uncoordinated at this large scale, consistent with regional noise driven by physiological processes tracking task difficulty [Garrett et al., 2014]. We explore the potential to gain additional insights into neurophysiological dynamics by applying these measures to previously acquired fMRI data in which 20 healthy adult individuals learned a set of novel finger sequences over the course of a 3‐day training regimen [Bassett et al., 2011]. Importantly, in this previous study, network flexibility was correlated with individual differences in learning. Thus, in this follow‐up methodological study, we are able to ask whether parsing a network's reconfiguration profiles into different types of flexible network changes provides a detailed picture of the cognitive dynamics underlying behavioral adaptation.

## METHODS AND MATERIALS

### Study Participants

The study consisted of 20 right‐handed participants that volunteered with informed consent in writing, in accordance with the Internal Review Board at the University of California, Santa Barbara. All scans were conducted at University of California, Santa Barbara. After exclusion criteria for task accuracy, incomplete scans, and abnormal MRI, 18 participants were retained for further statistical analysis. Study participants had little musical experience (less than 4 years with any one musical instrument), had normal vision, and no history of neurological disease or psychiatric disorders. All participants were paid for their participation.

### Motor Learning Task

Participants were placed in the MRI scanner with padding under their knees to maximize comfort and under the left forearm to minimize muscle strain when pressing buttons on the response box. To minimize head motion, padded wedges were also inserted between the participant and head coil of the MRI scanner. Participants performed a cued sequence production task, responding to visually cued sequences by generating responses using the 4 fingers (the thumb was excluded) of their non‐dominant (left) hand on a custom fiber‐optic response box. Visual cues were presented as a series of musical notes on a 4‐line music staff: the top line of the staff mapped to the leftmost key depressed with the pinkie finger and the bottom line of the staff mapped to the rightmost key depressed with the index finger. Each 12‐element note sequence contained three notes per line, which were randomly ordered without repetition and free of regularities such as trills (e.g., 121) and runs (e.g., 123). The number and order of sequence trials was identical for all participants.

A trial began with the presentation of a fixation signal, which was displayed for 2 s. The complete 12‐element sequence was presented immediately following the removal of the fixation, and participants were then instructed to respond as soon as possible. They were given a period of 8 s to type each sequence correctly. Participants trained on a set of 16 unique sequences, and there were three different levels of training exposure. Over the course of the three training sessions, three sequences—known as skilled sequences—were presented frequently, with 189 trials for each sequence. A second set of three sequences, termed familiar sequences, were presented for 30 trials each throughout training. A third set composed of 10 different sequences, known as novice sequences, were also presented; each novice sequence was presented 4–8 times during training. In this study, we focus exclusively on the skilled sequence blocks. Skilled and familiar sequences were practiced in blocks of 10 trials, so that 9 out of 10 trials were composed of the same sequence and 1 of the trials contained a novice sequence. If a sequence was reported correctly, then the notes were immediately removed from the screen and replaced with the fixation signal, which remained on the screen until the trial duration (8 s) was reached. If there were any incorrect movements, then the sequence was immediately replaced with the verbal cue INCORRECT and participants subsequently waited for the start of the next trial. Trials were separated with an inter‐trial interval (ITI) lasting between 0 s and 20 s, not including any time remaining from the previous trial. Following the completion of each block, feedback (lasting 12 s and serving as a rest) was presented that detailed the number of correct trials and the mean time that was taken to complete a sequence. Training epochs contained 40 trials (i.e., 4 blocks) and lasted a total of 345 scan repetition times (TRs), which took a total of 690 s. There were six scan epochs per training session (2,070 scan TRs). In total, each skilled sequence was presented 189 times over the course of training (18 scan epochs; 6,210 TRs).

To familiarize participants with the task, they were given a short series of warm up trials the day before the initial training session inside the scanner. Practice was also given in the scanner during the acquisition of the structural scans and just prior to the start of the first training‐session epoch. Stimulus presentation was controlled with MATLAB version 7.1 (Mathworks, Natick, MA) in conjunction with Cogent 2000 (Functional Imaging Laboratory, 2000). Key‐press responses and response times were collected using a fiber‐optic custom button box transducer that was connected to a digital response card (DAQCard‐6024e; National Instruments, Austin, TX).

In this study, we focus exclusively on the skilled sequence blocks. For further details, see [Bassett et al., 2011].

### Learning Rate Analysis

For each sequence, movement time (MT) was defined as the duration between the time of the first and last button press. Next, we computed the learning rate separately for each scan session, and for each sequence, and then we averaged these estimated rates across sequences. Specifically, the learning rate was computed by fitting a double exponential function to the MT as a function of the number of trials practiced during a single scan session and sequence [Rosenbaum, 2009; Schmidt and Lee, 1988] using a robust outlier correction in MATLAB (performed using fit.m function in the Curve Fitting Toolbox with option “Robust” and type “LAR”):

$$MT=D_{1}e^{-t\kappa }+D_{2}e^{-t\lambda }$$

where t is time, κ is the exponential drop‐off parameter (which we called the learning rate) used to describe the fast rate of improvement, λ is the exponential drop‐off parameter used to describe the slow, sustained rate of improvement, and $D_{1}$ and $D_{2}$ are real and positive constants. During the first session, the MT was relatively large, but as the subject became more familiar with the task, the MT decreased approximately exponentially. We therefore quantified learning rate by the exponential decay rate of MT as a function of trials practiced; the faster the decay rate, the quicker the learning. The magnitude of κ indicates the gradient of the learning slope where a sharper drop‐off in MT corresponds to individuals who are faster learners [Dayan and Cohen, 2011; Yarrow et al., 2009].

### Scanning Protocol

fMRI recordings were collected during each of the three training sessions using a 3.0 T Siemens Trio with a 12‐channel phased‐array head coil. For each functional run, a single‐shot echo planar imaging sequence that is sensitive to blood oxygen level dependent (BOLD) contrast was used to acquire 33 slices (3 mm thickness) per TR, with a TR of 2,000 ms, an echo time of 30 ms, a flip angle of 90°, a field of view of 192 mm, and a 64 × 64 acquisition matrix. Image preprocessing was performed using the Oxford Center for Functional Magnetic Resonance Imaging of the Brain (FMRIB) Software Library (FSL), and motion correction was performed using FMRIB's linear image registration tool. Images were high‐pass filtered with a 50s cutoff period. Spatial smoothing was performed using a kernel where full width at half maximum was 8 mm. Signals were normalized globally to account for transient fluctuations in intensity.

### Network Analysis

We constructed brain networks as graphs containing two types of elements: nodes and edges. Nodes represented brain regions derived from high resolution (T1) structural scans for each subject. These scans were divided into 112 regions based on the Harvard‐Oxford Atlas, a probabilistic atlas covering cortical and subcortical areas [Desikan et al., 2006]. Connections or edges between nodes represented the pairwise coherence of the average fMRI time series for a pair of brain regions [Bassett et al., 2011; Braun et al., 2015; Zhang et al., 2016].

Specifically, we used wavelet coherence (WTC), which identified areas in time‐frequency space where two time series co‐varied in the frequency band 0.06–0.12 Hz. We chose the WTC for reasons of statistical robustness. Wavelet decomposition is a method used to extract the portion of a signal that lies within a particular frequency band [Percival and Walden, 2000]. While conceptually similar to a band‐pass filter, a wavelet decomposition has several advantages in the context of fMRI BOLD signal time series including denoising [Fadili and Bullmore, 2004], robustness to outliers [Achard et al., 2006], and utility in null model construction [Pritchard et al., 2014]. Even more pertinent to the study here, because fMRI time series are long‐memory processes [Maxim et al., 2005; Wink et al., 2006], correlation, and coherence between two time series is not properly estimable from a statistical perspective [Beran, 1994], while such relationships between two wavelet coefficient times series *are* estimable [Achard et al., 2008; Gencay et al., 2001; Whitcher et al., 2000]. Here, we use wavelets to decompose the time series into a frequency band (0.06–0.12 Hz) that has previously been used to measure functional associations between low‐frequency components of the fMRI signal, and has marked utility in assessing task‐related functional connectivity [Bassett et al., 2011; Sun et al., 2004; Telesford et al., 2016]. We chose a measure of coherence over correlation between these time series because it is less sensitive to outliers [Devlin et al., 1975; Huber, 2004], and is independent of inter‐regional differences in the HRF, which can cause non‐trivial variations in a Pearson correlation coefficient that are independent of the underlying neural activity [Sun et al., 2004, 2007].

Collectively, these procedures resulted in a 112 × 112 weighted adjacency matrix with coherence values bounded between 0 and 1 for each functional connection or network edge.

### Multilayer Network Analysis

Most neuroimaging studies using brain networks utilize a static network analysis, which constructs networks using all functional data acquired in an entire scan session. In this study, we used a dynamic network analysis, where the functional data is subdivided into shorter time intervals (or windows), resulting in a series of networks representing the coherence between brain regions within each time window [Telesford et al., 2016]. Here, we extracted time series from each block of trials, treated each block as the temporal window of interest, and calculated the functional connectivity for each block. In this study, time series were subdivided into twenty‐five 160 s windows (2.67 min or 80 time points) for each scan session, thus representing temporal fluctuations on a time scale particularly relevant to the temporal scale of changes in behavior accompanying motor sequence training [Bassett et al., 2011]. Note that the high‐pass filter used above on the time series is justified given this time window length.

To better understand the temporal changes in network organization during training, we utilized a multilayer network approach to assess dynamics in community structure [Kivelä et al., 2014], representing changes in the functional modules recruited by the brain to perform the task. Community structure in a network indicates that nodes in a community are more interconnected with one another than they are with the rest of the network. This structure is often identified in multilayer networks using community detection algorithms such as the optimization of the modularity quality function [Bassett et al., 2011, 2013a, 2013a; Davison et al., 2015; Doron et al., 2012; Mantzaris et al., 2013]. Modularity maximization approaches [Fortunato, 2010; Newman, 2006a; Porter et al., 2009] can be used to find putative functional modules in the human brain [Bassett et al., 2011; Mucha et al., 2010].

The modularity quality function describes the partitioning of a network's nodes into communities via a comparison to a statistical null model [Newman, 2006a, 2006b; Newman and Girvan, 2004]. A generalization of the modularity quality function for multilayer networks can be defined as [Mucha et al., 2010]

$$Q=\frac{1}{2\mu }\sum_{ijlr}\left\{\left(A_{ijl}-\gamma_{l}P_{ijl}\right)\delta_{lr}+\delta_{ij}\omega_{jlr}\right\}\delta \left(g_{il},g_{jr}\right),$$

where *l* is the number of layers in the multilayer network, $A_{ijl}$ is the adjacency matrix, $P_{ijl}$ is the corresponding null model matrix given by the Newman‐Girvan null model defined as *k_i_k_j_*/2*m* where *m* is the average edge weight in the matrix, $\gamma_{l}$ is the structural resolution parameter, which defines the weight of intralayer connections (in this study $\gamma_{l}=1$), $g_{il}$ gives the community assignment of node i in layer l, $g_{jr}$ gives the community assignment of node j in layer r, and $\omega_{jlr}$ is the connection strength between nodes in consecutive layers (in this study $\omega_{jlr}=1$). Note that this *Q* value is also called the multilayer modularity index, or more simply the multilayer modularity. We use a Louvain‐like locally greedy algorithm [Blondel et al., 2008] to identify the partition of nodes into communities that maximizes the multilayer modularity. This optimization procedure yields a partition of brain regions into communities for each time window. This time‐dependent community assignment represents the evolution of putative functional modules in the brain as training occurs. As the community detection algorithm is non‐deterministic and susceptible to near degeneracies [Good et al., 2010], we optimized the multilayer modularity quality function 100 times for each temporal network [Bassett et al., 2013b].

In this study, we utilized a temporal window null model to determine statistical significance [Bassett et al., 2011, 2013b; Telesford et al., 2016]. The null model was constructed by permuting the ordering of windows in the network uniformly at random while preserving intralayer and interlayer connections. This randomized window model disrupts the temporal ordering of windows, which informs the stability of communities over time. For our analysis, a total of 100 randomized networks were generated for each subject. Afterward, the multilayer modularity quality function was optimized 100 times for each random network. These null models were used to determine the statistical significance of network dynamics using permutation testing, as discussed in greater detail in the next section.

From a neurophysiological perspective, the multilayer modularity quality function (and its maximization) allows us to identify changes in communities in functional brain networks over time. Such changes have previously been linked to changes in cognitive strategies during learning [Bassett et al., 2015], changes in excitatory/inhibitory balance during tasks requiring working memory performance [Braun et al., 2016], and regional differences in domain‐general *versus* domain‐specific processing [Fedorenko and Thompson‐Schill, 2014]. In comparison to applying a series of maximizations of the static (non‐multilayer) modularity quality function which makes the false assumption that patterns of functional connectivity in the brain in each time window are independent from one another, the multilayer modularity quality function hard‐codes the temporal dependence of functional connectivity patterns across time windows. Moreover, it provides tuning parameters to assess community structure in these time windows at varying topological scales and varying temporal scales, a capability that is particularly relevant for the study of neural markers of learning which can occur in local and distributed circuits differentially in early *versus* late learning [Bassett and Mattar, 2017].

### Measures of Network Dynamics

One important aspect of multilayer modularity is that it reveals changes in community assignment of brain regions over time, driven by dynamic patterns of functional connectivity. One way to describe this temporal variability of community structure is to measure *node flexibility*, which is defined as the average number of switches a node makes between communities over time [Bassett et al., 2011]. Network flexibility has been used in multiple studies as a method to describe core‐periphery dynamics in brain networks where nodes with low flexibility are considered to form a temporal core critical for task execution, while nodes with high flexibility are considered to form a temporal periphery which may play a supporting role in task performance [Bassett et al., 2013b].

**Figure 1:**
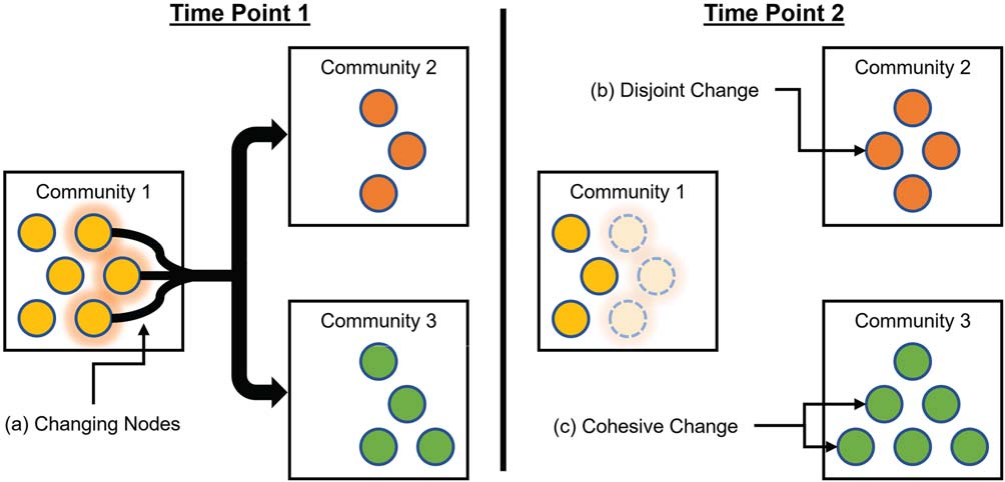
Schematic of cohesive and disjoint community changes. Dynamic community structure describes how nodes change across communities. (**a**) Nodes in Community 1 at Time Point 1 will shift from their community to different communities in Time Point 2. However, how these nodes move is unclear. (**b**) If a node moves from one community to another community independently from the movements of other nodes, this represents a disjoint *change*. (**c**) If a set of nodes moves as a group from one community to another, this represents a *cohesive change*. [Color figure can be viewed at http://wileyonlinelibrary.com]

Although node flexibility identifies the frequency that nodes change communities, it does not address the way in which nodes change communities. One approach for understanding the underlying network dynamics is to quantify node changes based on mutual *versus* independent changes (Fig. 1a). We define such quantities and refer to them as node cohesion and node disjointedness. Intuitively, **node disjointedness** describes how often a node changes communities *independently* from other nodes (Fig. 1b): that is, where a node moves from community *i* to community *j*, and no other nodes move from community *i* to community *j*. In contrast, **node cohesion** describes how often a node changes communities *mutually* with other nodes (Fig. 1c). Node disjointedness is defined by the number of times a node changes communities independently, divided by the number of times a node can change communities (which is equal to the number of time windows minus unity). In contrast, node cohesion measures community changes based on the pairwise changes between nodes, and is expressed as a cohesion matrix, where edge weight denotes the number of times a pair of nodes change to the same community together, divided by the number of times nodes can change communities.

A benefit of this matrix representation of the co‐switches of nodes from one community to another is that it facilitates the definition of additional matrix‐based measures. Perhaps the simplest—and the one we therefore focus on here—is akin to the notion of degree: **cohesion strength** represents the sum of row entries in the cohesion matrix, ignoring values along the diagonal. Higher values of cohesion strength indicate that nodes change communities often with other nodes; lower values of cohesion strength indicate that nodes change communities infrequently with other nodes. To gain some intuition for how these statistics may quantify different sorts of network changes, we consider the topic of splitting *versus* merging communities, and the relation of these behaviors to our measures of cohesion and disjointedness. If a community splits into two, then cohesion will be non‐zero, as a group of nodes is moving from old community A to new community B; a separate group of nodes is moving from old community A to new community C. Node disjointedness in this case is zero. In the case of merging communities, the behavior is similar: high cohesion, but zero disjointedness. In this study, we focus on cohesion strength and node disjointedness as they represent two forms of interactions that underlie node dynamics within a changing community structure. As the multilayer community detection algorithms used here are non‐deterministic (as discussed earlier in this Methods section), estimates for cohesion strength and node disjointedness were averaged across 100 optimizations of the modularity quality function.

### Tests and Procedures

Here, we briefly describe the analytical procedures and associated statistical testing methods that will be used in the Results section. To test for significant differences between dynamic network metrics (average cohesion strength and average disjointedness) in the true data and in the temporal network null model described in an earlier section, we used a two‐sample *t*‐test. To test for day‐to‐day variations in dynamic network metrics (cohesion strength and disjointedness), we used a one‐way repeated measures ANOVA in which one of the metrics was a dependent variable, and scan session was a repeated measure. To examine the degree of change in dynamic network metrics between consecutive days, we computed differences in average cohesion strength (or average disjointedness) for each participant between Day 1 and Day 2, and between Day 2 and Day 3. We tested whether the average difference between day pairs was statistically significant using a paired *t*‐test. Relationships between dynamic network metrics and learning rate across individuals was tested with a Pearson correlation coefficient.

In the main findings presented in this article, we fix two parameters in the multilayer community detection technique to their default values in the field of applied mathematics, which are a structural resolution parameter $\gamma_{l}=1$ and a temporal resolution parameter $\omega_{jlr}=1$. These values indicate an equal weighting of the true adjacency matrix and that of the random network null model, an assumption that is warranted if evidence supporting a different weighting is not available. However, to further ensure robustness of our results, we also report spatial distributions of dynamic community statistics (average cohesion strength and disjointedness) over a range of resolution parameters. Specifically, because tangible communities did not appear below *γ* = 0.91, we set a parameter space about the range equidistance from the default values of 1, for *γ* = [0.91 1.1] and *ω* = [0.91 1.1] at increments of 0.01. At each value of *γ* and *ω*, we performed a permutation test (10,000 permutations) between the values of the dynamic community statistics (average cohesion strength and disjointedness) at each node between Day 1 and Day 2 of training. We report brain regions that showed significant differences in graph metric values between Day 1 and Day 2 after controlling for type I errors using a false discovery rate (FDR) correction.

## RESULTS

### Significant Dependent and Independent Changes in Community Structure

**Figure 2:**
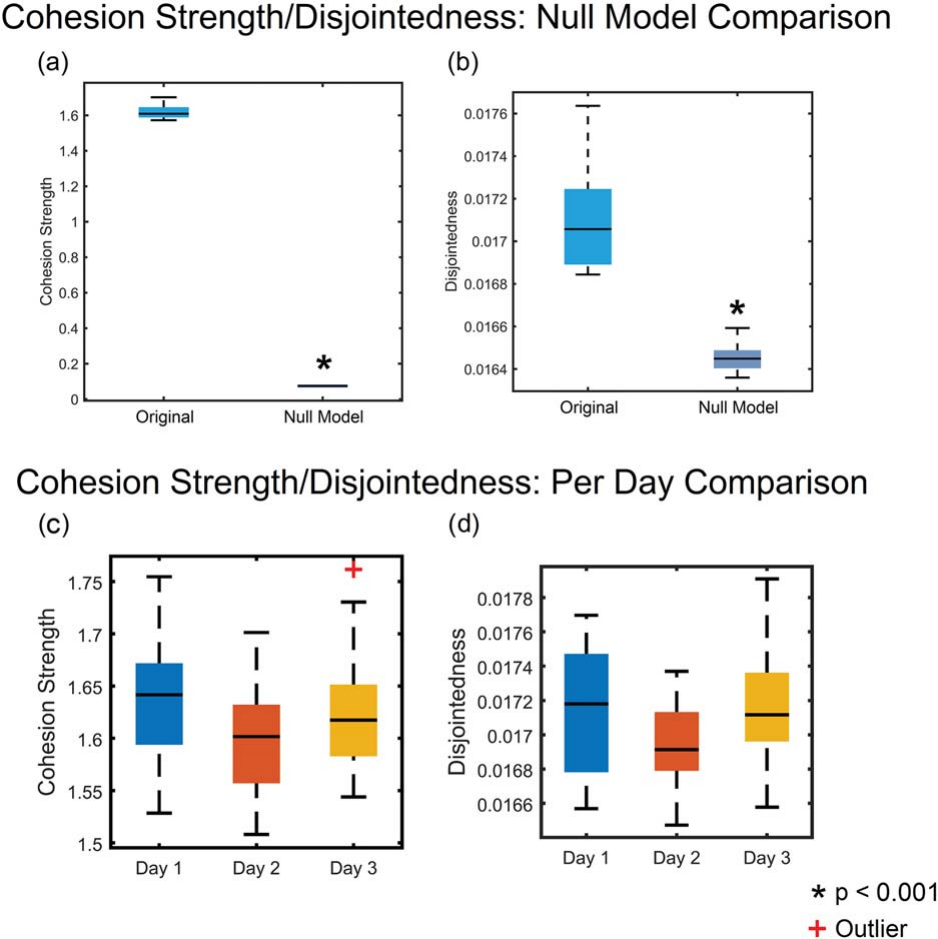
Box and whisker plot comparison of cohesion strength/disjointedness with null model and across days. An independent‐samples *t*‐test was conducted comparing (**a**) cohesion strength and (**b**) disjointedness to equivalent null model values averaged across all scan sessions. There was a significant difference between the average metric across days and the random window null model for cohesion strength, *t*(94) = 191.75, *P =* 7.08 × 10^−162^ and disjointedness, *t*(94) = 13.18, *P =* 8.37 × 10^−20^. A one‐way repeated measures ANOVA was conducted to compare the effect of scan session (day) on (**c**) cohesion strength and (**d**) disjointedness. There was no significant difference across days for cohesion strength *F*(2,15) = 1.55, *P* = 0.23. Likewise, there was no significant difference across days for disjointedness *F*(2,15) = 1.83, *P* = 0.18. An asterisk (*) indicates *P* < 0.001. The red “+” indicates an outlier. [Color figure can be viewed at http://wileyonlinelibrary.com]

After defining the new measures of network reconfiguration (cohesion strength and disjointedness), a natural first question is whether the human brain displays values for these statistics that would *not* be simply expected in random network null models. We addressed this question in the context of dynamic networks extracted from task‐based fMRI data acquired during motor skill learning (see Methods). Using an independent‐samples *t*‐test, we compared the empirically observed values of cohesion strength (averaged across all three scanning sessions) and of disjointedness (also averaged across sessions) to those observed in a random network null model. Scans from all sessions for each participant were grouped together into the “Original” group (*n* = 48) and compared to a group of their null model equivalents (*n* = 48). The equivalent null model for each scan session was based on the average across 100 optimizations of the modularity quality function. We chose the most stringent null model currently developed for multilayer functional networks in human neuroimaging, which is known as the temporal null model. This null model enabled us to test the null hypothesis that the empirically measured functional brain networks displayed dynamic community statistics that were no different than those expected when the order of time windows was permuted uniformly at random. We observed that average cohesion strength was significantly higher than expected in the temporal null model, *t*(94) = 191.75, *P* = 7.08 × 10^−162^ (Fig. 2a), suggesting that the brain displayed more temporally cohesive dynamics than expected. Similarly, average disjointedness was significantly higher than that expected in the temporal null model, *t*(94) = 13.18, *P* = 8.37 × 10^−20^ (Fig. 2b), suggesting that the brain also displayed more temporally transient dynamic than expected. Together, these two results point to a greater range of community dynamics (both cohesive and disjoint) in true functional brain networks in comparison to the null. See the Results section of the Supporting Information for assessments of the relationships between cohesion, disjointedness, and other measures of community structure.

The next natural question is whether the observed range of community dynamics was true in each scanning session separately, or whether it was observed on only one or two of the three days. To address this question, we performed a one‐way repeated measures ANOVA to estimate the effect of scan session (day) on cohesion strength and disjointedness. In general, we observed no significant effect of day on the dynamic community statistics. First, we observed no significant differences in cohesion strength across days: *F*(2,15) = 1.55, *P* = 0.23. Similarly, we observed no significant differences in disjointedness across days: *F*(2,15) = 1.83, *P* = 0.18 (Fig. 2c,d). From the set of results presented in this section, we conclude (i) that brain network community structure displays non‐trivial dynamics over the course of learning, as supported by the differences between the observed dynamics and those expected in null models, and (ii) that these dynamics are—on average over subjects—relatively consistent from the first day of training to the last day of training. The results also offer more specific insights into the types of reconfigurations in community structure that occur than is possible with network flexibility alone. Specifically, the reconfigurations are characterized both by an unexpectedly high rate of cohesive changes in a node's allegiance to modules, and also an unexpectedly high rate of independent changes in a node's allegiance to modules.

### Individual Differences in Dependent and Independent Changes in Community Structure

**Figure 3:**
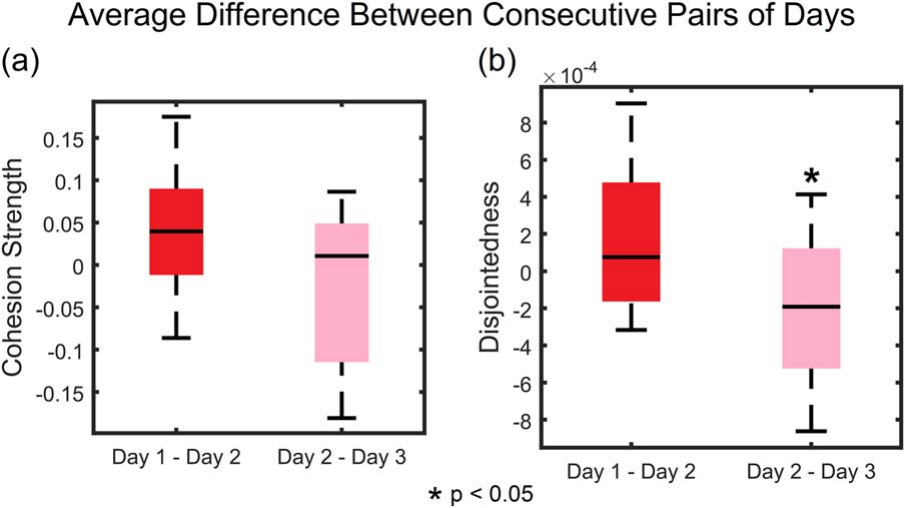
Comparison of average difference of dynamic graph metrics between consecutive pairs of days. A paired *t*‐test was used to compare the level of changes between pairs of days. (**a**) There were no significant differences in the changes between Day 1 and Day 2 compared to Day 2 and Day 3 for cohesion strength, *t*(15) = −1.50, *P* = 0.16. (**b**) In contrast, the changes between Day 1 and Day 2 were significantly higher than Day 2 and Day 3 for disjointedness, *t*(15) = −2.50, *P* = 0.02. [Color figure can be viewed at http://wileyonlinelibrary.com]

The analyses described above offer initial evidence supporting the notion that the brain shows a range of distinct community reconfiguration profiles during motor skill learning. However, the tests performed remained agnostic to the possibility that a single individual could show a decrease or increase in dynamic community statistics as they learned. To better account for individual differences in day‐to‐day changes in network dynamics (perhaps driven by individual differences in performance), we next examined the degree of change in network statistics between consecutive days. Specifically, we computed differences in dynamic community statistics for each participant between Day 1 and Day 2, and between Day 2 and Day 3. Then, we tested whether the average difference between day pairs was statistically significant using a paired *t*‐test. Although cohesion strength did not show significant differences between day pairs [*t*(15) = −1.4975, *P* = 0.16], disjointedness showed significantly higher changes between Day 1 and Day 2 than between Day 2 and Day 3 [*t*(15) = −2.50, *P* = 0.02] (Fig. 3). These results indicate that—at the level of individual participants—community changes between the first and second day are largely driven by independent changes in the affiliation of nodes (brain regions) to communities (putative functional modules supporting task performance).

### Dependent (and Not Independent) Changes in Community Structure Are Correlated With Individual Differences in Learning

We next sought to determine if these dynamic community statistics had predictive value in explaining individual differences in task performance. In a prior report utilizing this data, it was shown that network flexibility was correlated with individual differences in the learning rate [Bassett et al., 2011]. Here, we seek to determine whether these predictions were driven by dependent changes in community structure (as measured by cohesion strength) or independent changes in community structure (as measured by disjointedness).

**Figure 4:**
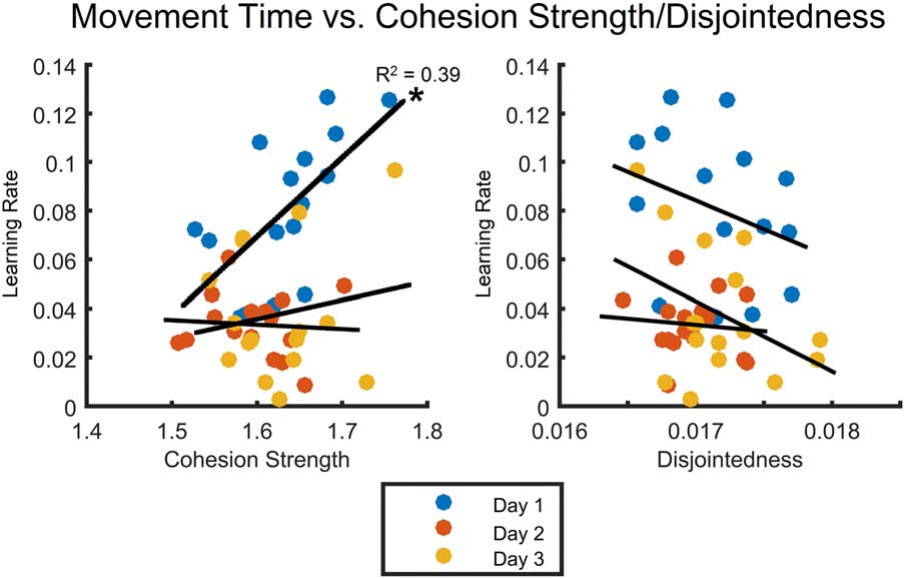
Learning rate (estimated as the exponential drop‐off in movement time) *versus* average cohesion strength and disjointedness. Average cohesion strength during the first scan session was the best predictor of performance (*R* ^2^ = 0.39, *P* = 0.01), while no significant relationships were observed on Day 2 (*R* ^2^ = 0.02, *P* = 5.19 × 10^−3^) and Day 3 (*R* ^2^ = 0.03, *P* = 0.53). Note that relationships between disjointedness and learning rate in each of the 3 days were not significant (>0.05, corrected for multiple comparisons using the Benjamini–Hochberg procedure with a false discovery rate correction, *q* = 0.05). [Color figure can be viewed at http://wileyonlinelibrary.com]

To address this question, we first quantified task performance by computing the learning rate for each individual. The learning rate is defined as the exponential drop‐off parameter of the MT as a function of trials; and MT is defined as the time between the first button press and the last button press of a 12‐note finger sequence (see Methods). If the learning rate is 0, then no behavioral change occurs, while if the learning rate is greater than 0, then behavioral change does occur. Larger values of learning rate indicate that the sequences were learned more quickly, while lower values of learning rate indicate that the sequences were learned more slowly. We observe in our data that learning rate differed over individuals and days of practice, covering a range from approximately 0 to approximately 0.13 (Fig. 4).

With these estimates of subject and scan‐specific learning rates, we asked whether individual differences in average cohesion strength or disjointedness was related to individual differences in learning. We observed that cohesion strength on the first day was the best predictor of learning rate (*R* ^2^ = 0.39, *P* = 0.01) (Fig. 4), while no significant relationships were observed on Day 2 (*R* ^2^ = 0.02, *P* = 5.19 × 10^−3^) and Day 3 (*R* ^2^ = 0.03, *P* = 0.53). For disjointedness, we observed no significant relationship for Day 1 (*R* ^2^ = 0.09, *P* = 0.26), Day 2 (*R* ^2^ = 9.54 × 10^−3^, *P* = 0.72), or Day 3 (*R* ^2^ = 0.16, *P* = 0.13). These results suggest that on the first day of practice, when the majority of the learning occurs for this task, functional brain network modules reconfigure in a cohesive manner: a set of brain regions that may have been functionally connected to one network module can change their activity profile and become functionally connected to another network module. Moreover, the greater the degree of cohesive movement, the better the learning. Note that relationships between disjointedness and learning rate in each of the 3 days were not significant (*P* > 0.05, corrected for multiple comparisons using the Benjamini–Hochberg procedure with a FDR correction, *q* = 0.05). Bridging these results with those of the previous section, we conclude that while disjointedness may account for the significant changes in network dynamics observed between the first and second day of task practice, it does not correlate with individual differences in learning rate.

### Regional Specificity of Dependent and Independent Changes in Community Structure

**Figure 5:**
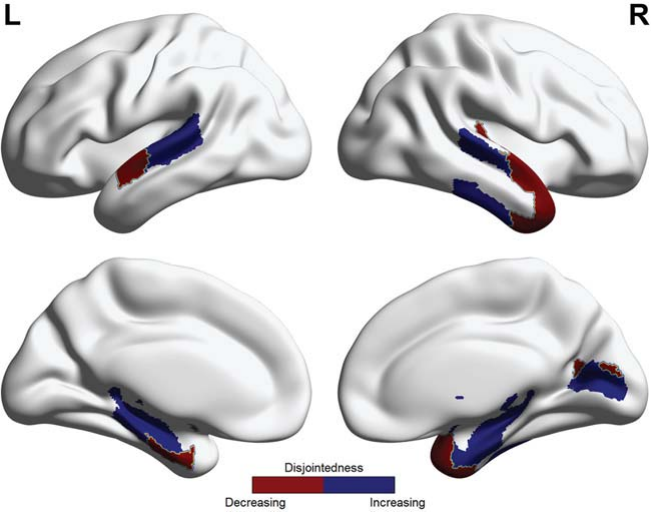
Statistically significant regions for differences in disjointedness between Day 1 and Day 2. Investigating disjointedness across the multilayer community detection parameter range *γ* = [0.91 1.1] and *ω* = [0.91 1.1], areas in the temporal lobe were found to be statistically significant consistently over the parameter range. Areas in red indicate disjointedness decreased between days, while areas in blue indicate disjointedness increased between days. [Color figure can be viewed at http://wileyonlinelibrary.com]

The results presented thus far have focused on dynamic community statistics averaged across the entire brain. We next turn to examining the anatomical location of cohesive movers and disjoint movers. Specifically, we seek to identify brain regions that show significant changes in dynamic community statistics from the first day of training to the second day of training, the time interval in which we observe the greatest learning. Then, we map these significant regions onto the surface of the brain. Importantly, these maps include sensitivity to network dynamics over a range of temporal and spatial scales [Sasai et al., 2011, 2014] by varying the spatial and temporal resolution parameters of the modularity quality function (see Methods). Notably, we observed no significant changes in cohesion strength in any nodes from Day 1 to Day 2; however, we did observe a set of brain regions that showed significant changes in disjointedness from Day 1 to Day 2 (Table 1). Areas that showed a significant increase in disjointedness from Day 1 to Day 2 included bilateral parahippocampal gyrus, hippocampus, brainstem and superior temporal gyrus. In contrast, areas that showed a significant decrease in disjointedness from Day 1 to Day 2 included the temporal pole, inferior temporal gyrus, planum polare, planum temporale, Heschl's gyrus, supercalcarine and intracalcarine cortex (Fig. 5). These changes, predominantly located in temporal cortex, are likely associated with neurophysiological processes related to sequence timing [Bueti et al., 2008] and higher‐order visual processing [Boggio et al., 2009; Doyon and Milner, 1991; Haxby et al., 2001], which become less of a focus of mental effort later in training.

**Table 1:** Regions showing significant difference between Day 1 and Day 2 of the learning task

Decreased disjointedness from Day 1 to Day 2		Increased disjointedness from Day 1 to Day 2	
Side	ROI	Side	ROI
R	Heschl's gyrus	L	Brainstem
R	Inferior temporal gyrus, anterior	R	Brainstem
L	Parahippocampal gyrus (superior to ROIs 34,35)	L	Hippocampus
R	Planum polare	R	Hippocampus
R	Supercalcarine cortex	R	Inferior temporal gyrus, posterior
L	Superior temporal gyrus, anterior	R	Intracalcarine cortex
R	Superior temporal gyrus, posterior	R	Parahippocampal gyrus (superior to ROIs 34,35)
R	Temporal fusiform cortex, anterior	L	Parahippocampal gyrus, posterior
R	Temporal pole	L	Planum temporale
		R	Superior temporal gyrus, anterior
		L	Superior temporal gyrus, posterior

Average disjointedness at each node was calculated across the multilayer community detection parameter range, *γ* = [0.91 1.1] and *ω* = [0.91 1.1]. Significant areas that demonstrated a consistent decrease or increase in disjointedness were located in hippocampal and temporal areas.

## DISCUSSION

The study of brain network dynamics represents an area of research that is increasingly important for an understanding of time‐varying fluctuations in brain function. In this study, we introduced a new concept for measuring dynamic changes in a network, specifically how nodes change their allegiance to putative functional modules over time. In this study, we established that the manner in which nodes change their allegiance is significantly different from the manner observed in comparable null model networks. These results underscore how changes in temporal ordering of functional connectivity patterns inform the stability of putative functional modules supporting cognitive processes during skill learning. In particular, the level of cohesive and disjointed changes in the allegiance of regions to putative functional modules offers finer‐scale information about the changes in network dynamics that accompany task performance [Telesford et al., 2016] as traditionally examined using network flexibility [Bassett et al., 2011]. More generally, the work offers methodological innovations that complement the growing battery of tools aimed at distilling neurophysiologically relevant changes in functional connectivity patterns over time [Hutchison et al., 2013a].

The findings presented in this article support a wider and growing literature on the time‐varying changes in functional brain network topology that can provide neural correlates of temporal variations in cognitive functions. These fluctuations can occur over long time periods, such as those observed in network topology over 18 months in recent longitudinal imaging studies [Shine et al., 2016b], where they correlate with temporal variations in self‐reported attention and mood [Betzel et al., 2017]. Fluctuations in functional brain network topology have also been observed over the course of task performance (for example, see [Braun et al., 2015, 2016; Bassett et al., 2011, 2015; Shine et al., 2016a]), where they explain temporal variations in task performance and are associated with dilations in pupil diameter, suggesting that ascending neuromodulatory systems may govern alterations in brain network state [Shine et al., 2016b]. In the context of learning studied here, the examination of changes in network topology enables us to track variations in functional connectivity that map to variations in learning rate, and to the learning process itself. Importantly, such insights are not possible with time‐invariant or static connectivity analyses [Hutchison et al., 2013a]. Moreover, recent studies also demonstrate that several insights provided by time‐varying functional connectivity (including predictions of individual differences in behavior) cannot be derived from patterns of neural activation as measured by a standard general linear model; for example, see [Bassett et al., 2015].

### Flexible Network Reconfiguration and Cognition

Prior work in multilayer network approaches has largely focused on network flexibility, which is a powerful metric for understanding changes in network communities during both task performance and during the resting state. When estimated across the whole brain, network flexibility is correlated with individual differences in motor skill learning [Bassett et al., 2011] and in reinforcement learning [Gerraty et al., 2016], changes within a single subject according to the subject's affect (positive vs. negative; aroused vs. not aroused) and level of fatigue [Betzel et al., 2017], and is modulated by the NMDA‐receptor antagonist Dextromethorphan suggesting its dependence on glutamate function [Braun et al., 2016]. When estimated in specific regions of the brain such as the frontal cortex, network flexibility has been shown to increase during high cognitive load in a 2‐back working memory task [Braun et al., 2015], and correlate with individual differences in cognitive flexibility [Bassett et al., 2015] and working memory accuracy [Braun et al., 2015]. These studies collectively suggest that the re‐arrangement of network communities—a proxy for putative functional modules supporting task performance—is an important dimension of dynamic functional connectivity [Hutchison et al., 2013b]. This claim is perhaps not surprising as many studies have demonstrated that the putative functional modules are consistently present in multiple cognitive states, across both healthy and diseased cohorts [Sporns and Betzel, 2016; Telesford et al., 2015], and across a wide range of developmental stages [Gu et al., 2015] and aging [Betzel et al., 2014].

On the backdrop of these general efforts in understanding reconfiguration of putative functional modules, it has been interesting to note that while flexibility appears to be a generally positive attribute of healthy cognitive function, excessive network reconfiguration appears to be a hallmark of psychiatric disease [Braun et al., 2016; Siebenhühner et al., 2013]. Together, these observations initially appear at odds with one another, yet they can be simply and intuitively reconciled with the notion that healthy individuals display one type of network reconfiguration, and people with mental illness may display other types of reconfiguration. Yet, confirming such a hypothesis requires that we build a set of tools to comprehensively describe different types of network reconfiguration. In this study, we take important steps toward distinguishing distinct types of network reconfiguration within the broader context of modular architecture, a fundamental organizational principle of brain structure and function [Sporns and Betzel, 2016]. Cohesive changes occur when groups of brain regions move together from one network module to another; disjoint changes occur when a brain region moves from one module to another without being accompanied by any other region. Distinguishing these two types of reconfiguration enables us to better understand the neurophysiological processes that accompany behavioral change, and they may also offer an informative toolset for similar questions about communication networks more generally. An interesting example comes from the evolution of social groups defined by co‐authored publications or phone call logs [Palla et al., 2007]. Here, stability of communities is a function of size: larger communities are sustained with faster rates of change in membership than smaller communities that survive longer when membership composition is largely static. Both social and brain networks represent dynamic systems in which changes in allegiance of nodes to modules are critical features of the system's stability and adaptability.

### The Role of Network Reconfiguration in Learning

In this specific motor learning task, network flexibility was previously found to be predictive of individual differences in learning rate, as measured by the exponential drop‐off in MT with number of trials practiced [Bassett et al., 2011]. Regions driving this predictive relationship were located in frontal, presupplementary motor, and temporal areas, which are known to play a supportive role in motor skill acquisition [Dayan and Cohen, 2011]. In the present study, we extend these earlier results by demonstrating that the specific type of flexibility occurring in putative functional modules is critically important in our understanding of network dynamics supporting human learning. In particular, we observed that cohesion strength on the first day of practice was significantly correlated with individual differences in learning rate, while disjointedness was not. From this observation, we extended our analysis to determine which regions may drive this effect; investigating the region‐specific relationship between cohesion strength with learning rate on Day 1, we observed significant correlations in the central opercular cortex, Heschl's gyrus, planum polare, planum temporale, and the superior temporal gyrus (*P* < 0.05, corrected for multiple comparisons using the Benjamini–Hochberg procedure with a FDR correction, *q* = 0.05). It is notable is how well these regions align with the regions showing significant changes in disjointedness between Day 1 and Day 2. With the exception of the central opercular cortex, all regions showing a significant correlation between cohesion strength and learning rate on Day 1 also demonstrated significant changes in disjointedness between Day 1 and Day 2 (Table 2).

**Table 2:** Regions showing significant correlations between cohesion strength and learning rate on Day 1

Significant correlation between cohesion strength and learning rate on Day 1		
Region	Left	Right
Central opercular cortex	*R* ^2^ = 0.45, *P* = 1.78 × 10^−3^	*R* ^2^ = 0.40, *P* = 3.13 × 10^−3^
Heschl's gyrus **	*R* ^2^ = 0.58, *P* = 8.93 × 10^−4^	*R* ^2^ = 0.37, *P* = 4.91 × 10^−3^
Planum polare **	*R* ^2^ = 0.40, *P* = 2.68 × 10^−3^	*R* ^2^ = 0.37, *P* = 4.46 × 10^−3^
Planum temporale **	*R* ^2^ = 0.65, *P* = 4.46 × 10^−4^	*R* ^2^ = 0.42, *P* = 2.23 × 10^−3^
Superior temporal gyrus, anterior division **		*R* ^2^ = 0.39, *P* = 4.02 × 10^−3^
Superior temporal gyrus, posterior division **	*R* ^2^ = 0.52, *P* = 1.34 × 10^−3^	*R* ^2^ = 0.39, *P* = 3.57 × 10^−3^

Regional cohesion strength per individual was found to have significant correlations in the Heschl's gyrus, planum polare, planum temporale, and the superior temporal gyrus (*P* < 0.05, corrected for multiple comparisons using the Benjamini–Hochberg procedure with a false discovery rate correction, *q* = 0.05). Many of these regions also demonstrated significant changes in disjointedness between Day 1 and Day 2 (highlighted with **).

These results suggest that mutual movement between *sets* of brain regions across existing modules is more conducive to learning than independent shifts in modular structure by regions. Moreover, these findings suggest that better outcomes in early learning require large perturbations in community structure. It will be interesting to see how these results are reflected in other learning tasks and to determine to what degree these large‐scale changes in functional connectivity map onto more local changes in functional activity [Wymbs and Grafton, 2015], or on to fine‐scale changes in the chunking of behavior [Wymbs et al., 2012]. Even outside the task, it may be interesting in future to determine whether an individual's predisposition to make cohesive changes may be predicted from baseline resting state function [Tavor et al., 2016] or from underlying white matter microstructure [Johansen‐Berg, 2010].

Apart from the study of individual differences, we also observed changes in the level of module reconfiguration that accompanied learning across all participants in the study. Specifically, we observed that differences in disjointedness between Day 1 and Day 2 were significantly higher than differences between Day 2 and Day 3, similar to previous findings using network flexibility [Bassett et al., 2011]. This pattern of changes suggests that disjointedness, like cohesion strength, also plays a significant role in the network dynamics that accompany learning. Interestingly, predominantly located in temporal and subcortical areas, these changes complement our earlier findings by demonstrating that while cohesive changes in network communities correlate with individual differences in learning, disjoint changes are consistent across individuals, and are a hallmark that distinguishes the two phases of skill acquisition: the swift rate of improvement observed in early learning *versus* the slow rate of improvement observed in late learning [Dayan and Cohen, 2011].

It is interesting to consider the question of whether different types of network reconfiguration can be thought of as different types of network noise [Garrett et al., 2013]; or whether certain network reconfiguration properties are more like noise than others. Intuitively, the notion of cohesiveness is perhaps less similar to notions of noise than disjointedness. Cohesiveness indicates a relatively low level of entropy, and the presence of low‐dimensional order in the system; disjointedness, conversely, indicates a relatively high level of entropy, and the presence of high‐dimensional dynamics in the system [Nakagawa et al., 2013]. Cohesiveness is unlikely to be driven by noise in the strictest sense of the term (either neurophysiological or artifact), and instead might indicate the presence of overlapping modules [Ball et al., 2011] or structural rearrangements [Johansen‐Berg, 2010]. Disjoint changes, by contrast, may be driven by neurophysiological noise [Mišić et al., 2010] consistent with an exploration of the landscape of possible strategies [Hills et al., 2015] required for early learning [Ishii et al., 2002].

### Methodological Considerations

Although these methods provide a promising approach for understanding network dynamics, some methodological considerations would be prudent to explore. The brain may display non‐trivial and neurophysiologically relevant network dynamics across multiple spatial and temporal scales [Sasai et al., 2011, 2014]. These scales can be explored in the context of dynamic community detection using the structural and temporal resolution parameters, *γ* and *ω*, respectively. Different choices of these parameters provide different insights into the spatial and temporal scales at which significant changes in network architecture are present [Telesford et al., 2016]. Here, we examine network reconfiguration across a range of scales by varying the values of both *γ* and *ω* around the classic default value of 1. This approach enables us to identify robust results consistent across parameter selection choices [Bassett et al., 2013a].

Another methodological consideration is choice of parcellation used to subdivide the brain into regions, as it is worth exploring the robustness of these measures at finer resolutions. A previous study comparing graph network properties across resolutions found robust preservation of network features [Hayasaka and Laurienti, 2010]. While network dynamics was not the focus of this study, their results suggest that a finer parcellation scheme may reveal inter‐regional dynamics not observed at coarser resolutions. Moreover, it is conceivable that further examination of network cohesiveness could lead to novel network parcellations based on dynamic interactions of nodes.

A third important consideration is the length of the time window used to assess the temporal variability in community structure present in functional connectivity patterns. Recent efforts have offered rules for best practices in choosing time windows that optimally balance statistical power and sensitivity to temporal change [Leonardi and Van De Ville, 2015], and that are maximally sensitive to individual variation in network dynamics [Telesford et al., 2016]. Consistent with prior work [Bassett et al., 2011], we use time windows lasting 2.67 min (80 time points), which provides enhanced statistical power to accurately estimate functional connectivity, while remaining sensitive to variations in functional connectivity patterns that accompany early learning.

Finally, we note that we have presented cohesion strength and disjointedness as two different measures, and as a complement to the previously defined measure of network flexibility. The interpretation of these measures therefore depends on their degree of independence. To assess any relation between the three metrics, we show in Supporting Information the Pearson correlation coefficients between disjointedness, cohesion strength, and flexibility. Briefly, we observe that there are no consistent patterns of correlations between any of these metrics over the course of the 3‐day experiment. These results suggest that there is no strict fundamental mathematical relation between these variables, and therefore that it is reasonable to study them as differential markers of neural function.

## FUTURE DIRECTIONS

In closing, we note that this work motivates a broader discussion on the development of methods to characterize the reconfiguration of brain networks in a variety of contexts. In particular, evidence suggests that flexibility itself, while positively correlated with many advantageous abilities (including cognitive flexibility, working memory, and learning), is also associated with psychiatric disorders such as schizophrenia [Braun et al., 2016]. These data suggest that other metrics of network reconfiguration may be helpful in distinguishing healthy flexible network properties from unhealthy flexible network properties. Our work begins to address this need. Moreover, to complement the development of additional descriptive statistics for brain network reconfiguration, the field would also likely benefit from a focus on developing mechanistic models of reconfiguration that would help us to better explain the neurophysiological processes driving optimal cognitive function.

## Supporting information

Supporting Information
