# Supplementary material for: Cohesive network reconfiguration accompanies extended training
Source: Hum Brain Mapp. 2017 Jun 24;38(9):4744–59. doi: 10.1002/hbm.23699 (PMC5554863; doi:10.1002/hbm.23699)
Supplement: Supplementary file 1 — Supporting Information [file HBM-38-4744-s001.doc]

**Supplementary Materials Document**

**I. Supplementary Results**

**I.a. Relation between dynamic network metrics**

Here, we explicitly report the Pearson correlation coefficients between disjointedness, cohesion, and flexibility. Briefly, we observe that there are no consistent patterns of correlations between any of these metrics over the course of the 3-day experiment. These results suggest that there is no strict fundamental mathematical relation between these variables, and therefore that it is reasonable to study them as differential markers of neural function.

Cohesion Strength vs. Disjointedness

|  | R^2^ | p-Value |
| --- | --- | --- |
| Day 1 | 0.01 | 0.76 |
| Day 2 | 0.00 | 0.81 |
| **Day 3** | **0.42** | ***0.007** |

Flexibility vs. Cohesion Strength

|  | R^2^ | p-Value |
| --- | --- | --- |
| Day 1 | 0.21 | 0.073 |
| **Day 2** | **0.68** | ***8.0x10^(-5)** |
| Day 3 | 0.17 | 0.11 |

Flexibility vs. Disjointedness

|  | R^2^ | p-Value |
| --- | --- | --- |
| Day 1 | **0.49** | ***0.003** |
| Day 2 | 0.18 | 0.10 |
| Day 3 | 0.05 | 0.40 |

* Denotes p < 0.05

**I.b. Relation between dynamic network metrics and other measures of community structure**

Here, we explicitly report the Pearson correlation coefficients between node disjointedness, node cohesion, and flexibility and statistics of community structure including number of modules, change in number of modules, and modularity Q. Briefly, we observe that the correlation between cohesion strength and disjointedness was significant on the third day, while flexibility and cohesion strength showed a significant correlation on the second day. Evaluating the number of communities and the change in number of communities, there were only significant correlations for cohesion strength with number of communities on the third day and cohesion strength with change in number of communities between the second and third day. It is tempting to speculate that these time-dependent changes in the relations between metrics could be indicative of different phases of learning; it would be interesting to explicitly test such a hypothesis in future empirical work.

Modularity

| **Flexibility vs. Modularity** | | |
| --- | --- | --- |
|  | R^2^ | p |
| **Day 1** | **-0.53** | *** 0.04** |
| Day 2 | 0.43 | 0.10 |
| Day 3 | 0.18 | 0.50 |

| **Cohesion Strength vs. Modularity** | | |
| --- | --- | --- |
|  | R^2^ | p |
| Day 1 | -0.50 | 0.05 |
| Day 2 | 0.19 | 0.48 |
| Day 3 | 0.22 | 0.42 |

| **Disjointedness vs. Modularity** | | |
| --- | --- | --- |
|  | R^2^ | p |
| Day 1 | -0.45 | 0.08 |
| Day 2 | 0.50 | 0.05 |
| Day 3 | 0.13 | 0.64 |

* Denotes p < 0.05

Flexibility vs. Number of Communities

|  | R^2^ | p-Value |
| --- | --- | --- |
| Day 1 | 0.02 | 0.62 |
| Day 2 | 0.02 | 0.61 |
| Day 3 | 0.14 | 0.15 |

Cohesion Strength vs. Number of Communities

|  | R^2^ | p-Value |
| --- | --- | --- |
| Day 1 | 0.08 | 0.29 |
| Day 2 | 0.11 | 0.21 |
| **Day 3** | **0.51** | ***0.0017** |

Disjointedness vs. Number of Communities

|  | R^2^ | p-Value |
| --- | --- | --- |
| Day 1 | 0.03 | 0.54 |
| Day 2 | 0.01 | 0.69 |
| Day 3 | 0.15 | 0.14 |

* Denotes p < 0.05

Change in Flexibility vs. Change in Number Communities

|  | R^2^ | p-Value |
| --- | --- | --- |
| Day 1 to Day 2 | 0.00 | 0.94 |
| Day 2 to Day 3 | 0.04 | 0.44 |

Change in Cohesion Strength vs. Change in Number Communities

|  | R^2^ | p-Value |
| --- | --- | --- |
| Day 1 to Day 2 | 0.02 | 0.62 |
| **Day 2 to Day 3** | **0.29** | ***0.030** |

Change in Disjointedness vs. Change in Number Communities

|  | R^2^ | p-Value |
| --- | --- | --- |
| Day 1 to Day 2 | 0.04 | 0.46 |
| Day 2 to Day 3 | 0.01 | 0.67 |

* Denotes p < 0.05

Absolute Change in Flexibility vs. Absolute Change in Number Communities

|  | R^2^ | p-Value |
| --- | --- | --- |
| Day 1 to Day 2 | 0.07 | 0.34 |
| Day 2 to Day 3 | 0.03 | 0.52 |

Absolute Change in Cohesion Strength vs. Absolute Change in Number Communities

|  | R^2^ | p-Value |
| --- | --- | --- |
| Day 1 to Day 2 | 0.01 | 0.73 |
| Day 2 to Day 3 | 0.00 | 0.82 |

Absolute Change in Disjointedness vs. Absolute Change in Number Communities

|  | R^2^ | p-Value |
| --- | --- | --- |
| Day 1 to Day 2 | 0.00 | 0.93 |
| Day 2 to Day 3 | 0.00 | 0.94 |
